# Herpetic viral dominance in pediatric meningitis and meningoencephalitis

**DOI:** 10.6026/973206300221448

**Published:** 2026-03-31

**Authors:** Pooja Mishra, Shweta Chauhan Pandey, Surjeet Kumar Shukla, Anusha Sharma, Shubham Prakash Singh, Manoj B Jais, Soumya Tiwari, Sanjib Gogoi, Vikash Yadav

**Affiliations:** 1 Department of Microbiology, Sunderlal Patwa Government Medical College (SPGMC), Mandsaur, Madhya Pradesh, India; 2 Department of Pediatrics, Birsa Munda Government Medical College, Shahdol, Madhya Pradesh, India; 3 Department of Microbiology, Dr Babasaheb Ambedkar Hospital, New Delhi, India; 4 Department of Microbiology, Lady Hardinge Medical College (LHMC), New Delhi, India; 5 Department of Pediatrics, LHMC and Associated Hospitals, New Delhi, India

**Keywords:** Viral meningitis, aseptic meningitis, herpetic meningitis, molecular detection meningitis, pediatric viral meningitis

## Abstract

Viral meningitis, caused by various viruses including *Enteroviruses*, Herpes simplex viruses and others, poses challenges in diagnosis due to its overlapping symptoms with other conditions. Therefore, it is of interest to evaluate the efficacy of RTPCR in detecting viral causes of meningitis in pediatric patients with suspected viral infections. Hence, a total of 62 CSF samples were analyzed, revealing a high prevalence of HSV2 and Parvovirus B19 among others. The study found RTPCR to be a sensitive and rapid method for identifying viral pathogens. This research advances knowledge by demonstrating RTPCR's utility in quickly identifying viral causes, aiding in timely and targeted treatment.

## Background:

Viral meningitis, also called aseptic meningitis, is caused by *Enteroviruses* (EVs), *Herpes simplex viruses, Influenza A viruses, Arboviruses*, etc. Viral meningitis is regarded as a slow and mild viral disease. In most studies, EVs are considered the primary cause of viral meningitis [1]. Viruses are the most common etiology for acute cases. Viral causes include *Enterovirus, Varicella zoster virus, Influenza A virus, Herpes simplex virus, *Mumps*, Measles, Parvovirus B19.*There are seasonal and regional variations in etiology, making the critical window for diagnosis and effective intervention often short [1, 2]. Viral meningitis affects mostly young children. The incidence decreases with age. In countries with high rates of immunization coverage, viral meningitis cases are more commonly seen than bacterial meningitis. *Vaccinations for Haemophilus influenza type B, Streptococcus pneumoniae and Neisseria * have led to significantly reduction in cases of bacterial meningitis. The incidence of viral meningitis has been estimated to range from 0.26 to 17 cases per 100000 people. In the United States, there are up to 75000 cases of *enteroviral meningitis* annually. In temperate climates, viral meningitis is most common in the summer and autumn months, while it is present year-round in tropical and subtropical areas [3]. HSV-1 is more commonly associated with sporadic encephalitis, while HSV-2 can cause benign recurrent viral meningitis. They can cause meningitis, in the absence of any herpetic genital lesions or a history of prior genital herpes virus infection. HSV reaches the central nervous system through the cranial nerves [3, 4]. LCMV is a rodent-borne virus, which spreads via inhalation. It is acquired through aerosolized urine or droppings. It can also transmit through vertical transmission. It is more common in winter and early spring season.

*Mumps* was previously a common cause of viral meningitis in the United States but has decreased recently due to implementation of measles, *Mumps* and rubella (MMR) vaccination policy [5, 6]. Infectious encephalitis can be due to viral, bacterial, fungal, protozoal or helminthic pathogens. The etiological agents causing encephalitis in many cases still remain unknown. Viruses are the most prevalent identified cause, accounting for about 70% of confirmed cases of encephalitis. In the United States, the most common causes of viral encephalitis are *herpes simplex virus (HSV), West Nile virus* and *Enteroviruses*. Some of the other viral etiologic agents include* Varicella-zoster virus, Epstein-Barr virus (EBV), Cytomegalovirus (CMV), Human Herpes virus type 6 and 7, Measles virus, Mumps virus, Rubella virus*. Other rare causes viral agents includes * vSt. Louis virus, Eastern equine virus, Western equine virus, Dengue virus and Rabies virus* [7, 8]. As all the viruses cannot be cultivated clinical signs, disease history and physical examinations constitute the potential diagnostic tools for paediatric viral meningitis. However clinical symptoms are almost similar in their phenotypic presentation in every case, despite of different viral etiology. Serological assays for viral antigen detection are proven efficient tool to diagnose viral meningitis but, PCR is considered as the gold standard test for viral meningitis detection. It detects viral nucleic acid with the highest sensitivity rates [1, 2]. Molecular tools are becoming the methods of choice for many laboratories and have improved public health measures because they allow multiple pathogens to be rapidly detected simultaneously. World Health Organisation [WHO] recommends the use of PCR in the testing of pathogens, from suspected cases of meningitis/meningoencephalitis. Major challenges remain, however, in the deployment of these assays in low- and middle-income countries, due to the variability of laboratory capabilities, shortage of trained laboratory personnel and challenges in procurement of reagents and equipment [9, 10-11]. Therefore, it is of interest to describe the challenges in deploying PCR assays for viral meningitis detection in low- and middle-income countries, as well as the potential benefits of molecular diagnostics in improving public health measures.

## Materials and Methods:

This cross-sectional of Microbiology, observation study was conducted in the Department Lady Hardinge Medical College, New Delhi in collaboration with the Department of Pediatrics, Smt. Sucheta Kripalani and Kalawati Saran childrens Hospital, New Delhi between November 2022 and February 2024. Detailed demographic and clinical details of the cases were entered in a pre-approved proforma. Total 62 CSF sample were analyzed during the study duration. Enrolled patients were >1 month to <18 years of age group i.e., pediatric age group. The CSF sample was transported to the Microbiology laboratory at the earliest in sterile leak-proof container after lumber puncture, for real time multiplex PCR molecular viral detection. Panel information - Nucleic acid amplification assay was used for qualitative detection and differentiation of different viral pathogens

## Viral panel:

*Enterovirus, Epstein bar virus, Herpes simplex virus1 and 2, Human adenovirus, Human cytomegalovirus, Human herpes 6 and 7, Human paraechovirus, Human parvovirusB19, Mumpsvirus, Varicella zoster virus*

## Results:

Demographic details: The mean age of the study group was 3.38 [±3.78] years. Our study shows a male preponderance with an M: F ratio of 1.58:1. Of the study participants, 61.2% (38/62) were males and 38.7% (24/62) were females. The most common clinical features noted during the study were fever (96.7% [60/62]), altered sensorium (67.7% [42/62]), abnormal body movements (56.4% [35/62]) and meningeal signs (29% [18/62]). Viral etiological profile detected by RTPCR: Out of 62 samples, 39 cases (62.9%) were detected with viral infection by RTPCR. Among the detected viral agents, HSV-2 was most commonly isolated with a 50% (31/62) positivity rate, followed by Parvovirus B19 (16.1% [10/62]), HH6 (4.8% [3/62]), HH7 (4.8% [3/62]), CMV (3.22% [2/62]), EBV (3.22% [2/62]) and HSV1 (3.22% [2/62]) as shown in Table 1 and Figure 1 (see PDF. The demographic and clinical findings of the study are summarized in Table 2 (see PDF), which presents the age-wise distribution of the etiological profile among the pediatric population. The viral etiological profile detected by RTPCR, along with the associated CSF and blood parameters, is further illustrated in Tables 3 and 4 (see PDF). Additionally, Table 5 (see PDF) shows the correlogram of viral RTPCR positivity with various laboratory parameters of CSF, providing insight into the relationships between viral presence and CSF characteristics. Similarly, Table 6 (see PDF) highlights the correlogram of viral RTPCR positivity with various blood laboratory parameters, offering further correlations between viral positivity and blood markers such as TLC and CRP. These tables collectively offer a comprehensive view of the clinical and laboratory parameters associated with viral meningitis and encephalitis in the paediatric population. CSF parameters of viral RTPCR-positive cases are presented in terms of median range, IQR (interquartile range), minimum and maximum values observed during the study. The following parameters were considered abnormal: CSF WBC cell count >5 cells / µL, >50% neutrophils, >25% lymphocytes, CSF sugar <40 mg/dL and CSF protein >40 mg/dL. These findings are presented in Table 3 (see PDF). The cytological profile of blood parameters for viral RTPCR-positive cases is presented in Table 4 (see PDF), showing median range, IQR, minimum and maximum values. Normal values were defined as TLC within 4000-11000/cumm, differential neutrophil count within 40%-71%, differential lymphocyte count within 18%-46% and CRP >7 mg/dL as abnormal. Statistical analysis revealed no statistically significant correlation between viral RTPCR positivity and various CSF laboratory parameters. However, a negative correlation between CRP and viral RTPCR positivity was found (r = -0.27, p = 0.032). The viral etiology detected by RTPCR is illustrated in Figure 1 (see PDF) . The distribution of viral agents in the 1m-6m age group is shown in Figure 2 (see PDF), while Figure 3 (see PDF) depicts the etiological profile in the 6m-12m age group. The 12m-24m age group is represented in Figure 4 (see PDF), followed by the 24m-60m age group in Figure 5 (see PDF), and finally, Figure 6 (see PDF) illustrates the etiological profile in the 60m-180m age group.

## Discussion:

Viral etiological profile was studied through RTPCR. Out of 62 samples 39 cases were positive for viral infection (62.9%). Ramamurthy *et al.* [12] reported 60% viral detection through RTPCR. HSV2 was the most common viral infection 19.35% (12/62) detected among paediatric age group. Various researchers have reported 10%-20% positivity rate for HSV2 in their studies. Leli *et al.* (2019) [13] reported 20% positive cases of HSV2 in his study. Study demonstrated the dominance of HSV2 infection among <2year paediatric age group (20/31). HSV virus is mainly responsible for meningitis due to recurrent or reactivation of virus due to low immunity or other comorbid conditions. HSV is reported to be commonly affecting, infants age group as seen in other studies [14, 15- 16]. In our study among herpes virus family HSV2 was found most commonly followed by HH6, HH7, CMV, EBV and HSV1. HSV2 was most commonly found in <2 years age group. Kumar *et al.* [71] reported Herpes simplex virus (31.50%) was the most common virus detected in <5 years age group, followed by Adenovirus (10.95%), Parvovirus (2.73%), Japanese encephalitis virus (1.36%), Enterovirus (1.36%) and Epstein-Barr virus (1.36%). Similarly, Aronson *et al.* [18] reported Herpes simplex virus was the most common cause of viral encephalitis. HH6 is also reported as a cause of meningitis/meningoencephalitis. Parvovirus B19 infection was second most common infection 20.96% (13/62) among viruses detected through RTPCR. It was most commonly detected among >1yr age group 16.1% (10/62). Douvoyiannis *et al.* [19] reported 31% (5/16) cases of ParvoB19 in CSF of children. Variation in positivity rate is seen due to difference in age group profile as they included all age groups including pediatric population Faraj *et al.* [20]. Barah *et al.* [21] documented 38.8% Parvovirus B19 related encephalopathy cases. As Indian studies related to molecular detection of Parvo B19 infection are limited hence further studies are required to support these findings.

## Conclusion:

RTPCR is a highly useful diagnostic tool for detecting low quantities of unviable organisms with high sensitivity, requiring only a small CSF sample. It provides rapid pathogen identification, allowing for targeted antibiotic therapy and reducing unnecessary antimicrobial exposure. The implementation of multiplex RTPCR in routine diagnostics can complement culture techniques; improve detection of viral meningitis and aid in early diagnosis, ultimately reducing mortality and morbidity in pediatric patients.
